# It’s No Has Bean: A Review of the Effects of White Kidney Bean Extract on Body Composition and Metabolic Health

**DOI:** 10.3390/nu12051398

**Published:** 2020-05-13

**Authors:** Ruth Nolan, Oliver M. Shannon, Natassia Robinson, Abraham Joel, David Houghton, Fiona C. Malcomson

**Affiliations:** 1 Human Nutrition Research Centre, Population Health Sciences Institute, Newcastle University, Newcastle upon Tyne NE2 4HH, UK; Ruth.Nolan@newcastle.ac.uk (R.N.); Oliver.Shannon@newcastle.ac.uk (O.M.S.); Aj.Joel@newcastle.ac.uk (A.J.); 2 School of Agriculture and Food Science, University College Dublin, 4 Dublin, Ireland; 3 Biosciences Institute, Newcastle University, Newcastle upon Tyne NE2 4HH, UK; N.Robinson5@newcastle.ac.uk; 4 Wellcome Centre for Mitochondrial Research, Translational and Clinical Research Institute, Newcastle University, Newcastle upon Tyne NE2 4HH, UK; david.houghton@ncl.ac.uk

**Keywords:** white kidney beans, metabolic health, obesity, body composition, α-amylase, *Phaseolus vulgaris* L.

## Abstract

The rising prevalence of overweight and obesity is a global concern, increasing the risk of numerous non-communicable diseases and reducing quality of life. A healthy diet and exercise remain the cornerstone treatments for obesity. However, adherence rates can be low and the effectiveness of these interventions is often less than anticipated, due to compensatory changes in other aspects of the energy balance equation. Whilst some alternative weight-loss therapies are available, these strategies are often associated with side effects and are expensive. An alternative or adjunct to traditional weight-loss approaches may be the use of bioactive compounds extracted from food sources, which can be incorporated into habitual diet with a low cost and minimal burden. One product which has attracted attention in this regard is white kidney bean extract (WKBE), which has been suggested to inhibit the enzyme α-amylase, limiting carbohydrate digestion and absorption with small but potentially meaningful attendant beneficial effects on body weight and metabolic health. In this review, drawing evidence from both human and animal studies, we discuss the current evidence around the effects of WKBE on body composition and metabolic health. In addition, we discuss evidence on the safety of this supplement and explore potential directions for future research.

## 1. Introduction

The world is currently facing an obesity pandemic, with the global prevalence of this condition tripling since 1975 [1]. In 2016, more than 1.9 billion adults worldwide (39%) were overweight and, of those individuals, 650 million (13%) were obese [2]. Childhood obesity is also increasing, with 41 million children recorded as overweight or obese in 2016 [2]. The burgeoning prevalence of obesity and its co-morbidities—which include type 2 diabetes, cardiovascular disease (CVD) and certain cancers [3,4]—place a substantial burden on public health services and the economy at large, costing the National Health Service (NHS) of the United Kingdom over £6 billion in 2015 [5]. Overweight and obesity were responsible for ~4 million deaths (7.2% of total deaths) globally in 2015 [6]. Furthermore, overweight and obesity, which were previously considered a problem exclusive to high-income countries, are now dramatically on the rise in low-income countries. Consequently, these countries are now not only facing concerns associated with malnutrition but also those associated with obesity [2].

Overweight and obesity result from a chronic positive energy imbalance, whereby energy intake exceeds energy expenditure [7]. The primary focus of obesity treatment is weight loss, which can be achieved by different strategies, including lifestyle (i.e., diet and exercise), pharmacological or surgical interventions [8,9]. Lifestyle interventions induce a negative energy balance by reducing energy intake (through dietary intervention) and/or increasing energy expenditure (through increased physical activity). The combination of dietary intervention and increased physical activity has been shown to have additive effects, reducing body weight and body fat and increasing fat-free mass compared with diet alone [10,11]. The implementation of lifestyle interventions can be difficult, as they rely on participant compliance, and long-term adherence and weight-loss maintenance is challenging [12]. In addition, lifestyle interventions frequently report smaller than anticipated reductions in body fat and body weight—an effect that can be explained by compensatory changes in other aspects of the energy balance equation. For example, exercise-induced increases in energy expenditure may be partly mitigated by exercise, stimulating an increase in energy intake and/or decreasing non-exercise physical activity [13,14].

Pharmacological agents present a potential strategy to aid weight loss alongside lifestyle interventions. For example, Orlistat, which inhibits pancreatic and gastric lipases, reduces the absorption of fat in the intestine and induces weight loss, however its use is limited due to associated side effects [15]. Furthermore, several other pharmacological treatments have been withdrawn from the market due to adverse effects, suggesting that pharmacological management of weight loss on its own is difficult. For example, Rimonabant was linked with an increased risk of psychiatric events including depression, whilst Fenluramine was demonstrated to increase the risk of CVD [16,17]. Surgical interventions (i.e., bariatric surgery), usually for people with severe or complicated obesity where lifestyle and pharmacological interventions have been unsuccessful [18], can achieve dramatic weight loss. However, surgery is not a cure for obesity on its own, and a commitment to permanent lifestyle and dietary changes is necessary post-surgery [19]. Furthermore, in addition to being expensive, surgical procedures are associated with risks and side effects such as malnutrition, resulting from the compromised gastrointestinal (GI) absorption of vitamins and minerals from food [20,21].

Interventions which can be used as an alternative or adjunct to traditional weight-loss strategies could be highly valuable to add to the ‘box of tools’ available to prevent or treat obesity. In this regard, bioactive compounds extracted from food sources, which can be incorporated into habitual diet, have shown some promise. Positive features of these food extracts include that they are inexpensive, can be incorporated into habitual diet with a minimal burden and are typically associated with a low risk of adverse events.

## 2. White Kidney Bean Extract

Alpha-amylase (α-amylase) is an enzyme that has a major role in carbohydrate metabolism [22], catalysing the hydrolysis of the α-(1,4) glycosidic linkages in starch and other oligosaccharides [23]. This may represent a potential mechanistic target for interventions seeking to inhibit carbohydrate uptake in the body. Indeed, bioactive ingredients that inhibit catabolic enzymes (i.e., amylase and glucosidase) are becoming increasingly popular [24], with potential applications for both weight loss and metabolic health.

White kidney beans (*Phaseolus vulgaris* L.) possess three isoforms of α-amylase inhibitors—α-AI1, α-AI2, α-AIL [25], with the α-AI1 isoform shown to inhibit amylase activity in mammals [26]. The structure of α-AI1 is a classic lectin fold, containing no α-helices and a plethora of antiparallel β-sheets [23]. α-AI1 has no carbohydrate-binding abilities due to the complete absence of surface carbohydrate-binding loops on its three-dimensional structure [26]. Functioning optimally within a pH range of 4.5–5.5 and a temperature range of 22–37 °C [26]. α-AI1 has been suggested to interact with pancreatic α -amylase at its active site, competitively inhibiting its binding with carbohydrate [26]. α-AI1 can block the enzyme’s substrate-reducing end and obstruct the non-substrate-reducing end through a steric hindrance process, targeting all catalytically-competent components of the enzyme [22]. Several commercially-available white kidney bean extracts (WKBEs) now exist which reportedly contain high quantities of white kidney bean-derived α-amylase inhibitors, typically available in powder form, allowing their encapsulation or incorporation into drinks and foods (for further information on the preparation of these extracts, see [26]). These supplements claim to reduce carbohydrate absorption and digestion via their α-amylase inhibitory properties, with attendant beneficial effects on body weight and metabolic health. Whilst this has been the subject of two meta-analyses, one was published almost ten years ago [27] and the other focussed on a specific brand (Phase 2) [28], and other forms of WKBE might be differentially effective. Therefore, in the following section, the evidence for and against these claims is reviewed.

## 3. Evidence from Animal Studies

### 3.1. Effects of WKBE on Body Weight and Composition

A number of animal studies, with intervention durations ranging from 14 to 238 days, have explored the effects of WKBE on body weight and composition (Table 1). Although not a universal finding [29,30,31], several studies have demonstrated significant reductions in body weight or reduced weight gain following the administration of WKBE versus a control [32,33,34,35,36,37]. For example, Song et al. [37] reported a significant attenuation in weight gain and reduced visceral fat in mice after 98 days of supplementation with 50 mg/kg/d WKBE alongside a high-fat diet when compared with a high-fat diet alone. In a similar study, Micheli et al. [34] explored the effects of WKBE on body weight and metabolic health in male C57BL/6 mice with metabolic syndrome induced by the prolonged (133 days) administration of a high-fat diet. Following 77 days consuming a high-fat diet, the animals were provided with WKBE (500 mg/kg/d), metformin (100 mg/kg/d) or atorvastatin (10 mg/kg/d)—drugs traditionally used for the management of hyperglycaemia and hypercholesterolemia, respectively—or the control. Mice consuming a normal diet had a significantly lower weight gain compared with all mice consuming a high-fat diet. However, during the administration of treatments (days 84–133), the mice supplemented with WKBE gained significantly less weight compared with the mice receiving the control. There was also a tendency towards a reduced body weight gain in metformin-treated mice, although this didn’t attain statistical significance.

Interestingly, recent findings from Shi et al. [32] suggest that the anti-obesity effects of WKBE may be both dose- and duration-dependent. The authors reported a significant reduction in body weight in male Sprague Dawley rats receiving the largest dose of WKBE (1.5% addition) for 42 days. However, after 70 days of supplementation, both the moderate (1.0% addition) and high dose showed significant reductions in body weight compared with the controls. Conversely, a low dose of WKBE (0.5% addition) did not affect body weight at 42 and 70 days of supplementation. In addition to overall weight loss, both moderate and high doses of WKBE reduced intra-abdominal fat accumulation and improved blood lipid profiles after 70 days supplementation—effects that may have important implications for metabolic health. Mechanistically, rats receiving the WKBE showed a lower food efficiency ratio, suggesting that they derived less energy from the food, which could be due to the reduced digestion and absorption of starch. Interestingly, the food intake was also lower in rats receiving WKBE, suggesting a potential anorexigenic effect of WKBE. A reduced food consumption consequent to WKBE supplementation has been substantiated by some [35,36] but not all investigations in this area [32,33,34,38,39]. Others have also identified potential alterations in gut microbiota composition and bile acid content consequent to WKBE consumption, which might play a role in the anti-obesity effects of this supplement [32,33,37], although further research is needed to substantiate this hypothesis.

Despite the promising findings noted above, not all studies have reported a reduction in body weight/reduced weight gain in animals supplemented with WKBE [29,30,31]. Further research is therefore warranted to elucidate specific factors which may account for the disparate findings reported in the literature, which could include the activity, purity, dose and duration of the supplement administered [32]; the health status and type or breed of animals studied [40]; and the quality of the research (e.g., the presence/nature of a control group, sample size, blinding of researchers) [41]. In addition, whilst animal model investigations provide useful insight into the potential effects of WKBE on body composition, it is important to consider how well these findings translate into humans, especially when considering a condition such as obesity, which has a complex and multifaceted aetiology. This will be explored in the following section.

### 3.2. Effects of WKBE on Cardiometabolic Markers and the Gut Microbiota

#### 3.2.1. Blood Markers

Studies investigating the impact of WKBE on blood biochemistry have primarily focused on blood glucose, triglycerides and cholesterol. In both mice and rats supplemented with a variety of different WKBEs and administered doses between 500 mg/kg and 4 g/kg, improvements in blood glucose have been reported when compared with the control groups [31,34,35,36,40,42]. Despite reductions in blood glucose, Tormo and colleagues did not observe accompanying improvements in plasma insulin levels [35,36]. In a diet-induced mouse model of obesity, WKBE reduced the circulating concentrations of triglycerides, total cholesterol and Low-Density Lipoprotein (LDL) when compared with mice given a high-fat diet alone [37]. These findings were accompanied by significant reductions in serum glucose, insulin and Homeostatic Model Assessment of Insulin Resistance (HOMA-IR), and a reduced area under the curve following an oral glucose tolerance test [37]. In addition, Micheli et al. [34] reported a significant reduction in insulin, total cholesterol, LDL and circulating triglycerides in the WKBE group when compared with the high-fat control group. Similarly, induced dyslipidaemia increased the cholesterol and triglyceride levels in obese rats, however, following 70 days on a medium and high dose of WKBE, the triglycerides and LDL reduced significantly [32]. In the latter two studies, a trend for improvements in total cholesterol and High-Density Lipoprotein (HDL) was also observed [32,34].

#### 3.2.2. Gut Microbiota Composition and Metabolism

The gut microbiota has attracted considerable attention in recent years as a potentially tractable mechanistic target for lifestyle interventions seeking to improve health. Although there have been no studies conducted in humans, animal investigations provide tentative evidence for a beneficial effect of WKBE on gut microbiota composition. Compared with mice fed a high-fat diet alone for 98 days, WKBE significantly reduced the relative abundance of Firmicutes; increased the relative abundance of Verrucomicrobia and Actinobacteria at the phylum level; and increased the relative abundance of the health-associated bacteria *Bifidobacterium, Lactobacillus* and *Akkermansia* at the *genus* level [37]. Similar findings were also reported in a later study by Neil et al. [33], who specifically sequenced Firmicutes and Bacteroidetes phyla and *Akkermansia Muciniphila* (*A. muciniphila)* at the *genus* level in the caecum of mice fed a high-fat diet with or without WKBE. A significant reduction in the Firmicutes/Bacteroidetes ratio and a 473-fold increase in *A. muciniphila* were observed in the treatment mice, both of which have been associated with health benefits—for example, greater levels of *A. muciniphila* have been associated with improved metabolic markers and a reduction in obesity and inflammation [43].

Recently, Shi et al. [32] sequenced the 16S rRNA gene to investigate the effects of WKBE on the gut microbiota, including composition and diversity metrics. A high-fat diet led to the dysbiosis of the gut microbiota, including a reduction in the indices of diversity, abundance, evenness and richness—such as α– and β-diversity and Shannon Index—for obese rats. The high dose of WKBE (1.5%) prevented the reduction in microbial composition diversity (β-diversity) induced by the high-fat diet. Specifically, at the phylum level, the relative abundance of Firmicutes and Proteobacteria decreased and Actinobacteria and Bacteroidetes increased when compared with obese rats not fed WKBE. At the *genus* and operational taxonomic level (OTU), WKBE increased the relative abundance of *Akkermansia* and altered 36 OTUs, 30 of which differed significantly from rats with high-fat diet-induced obesity. Interestingly, the OTUs enriched following WKBE supplementation were associated with short-chain fatty acid (SCFA)-producing bacteria, such as *Butyricoccus*. Such SCFA-producing bacteria have been associated with protection against obesity and related metabolic diseases, for example by counteracting obesity-induced inflammation [44]. Fittingly, total SCFA concentrations (driven predominantly by increases in acetate and propionic acid) in the colonic contents of rats fed medium and high doses of WKBE were significantly greater than those measured in the control and obese groups. In addition, some of the differential OTUs in the WKBE group correlated with obesity indices; for example, an inverse relationship was observed between six OTUs belonging to *Bacteroides* and body weight and intra-abdominal fat accumulation. Interestingly, a reduction in Bacteroidetes has been associated with obesity, and weight loss following a low-calorie diet has been shown to increase the proportion of Bacteroidetes in human adults [45].

#### 3.2.3. Oxidative Stress Markers

Obesity-induced oxidative stress is a key mediator of the effects of obesity on health-related outcomes and the development of co-morbidities [46]. Furthermore, oxidative stress is implicated in the pathogenesis of obesity and is modulated by risk factors for obesity, such as dietary intake and the consumption of bioactive compounds [47]. A limited number of animal studies have investigated the effects of WKBE administration on the markers of oxidative stress. In the study by Micheli et al. [34] a high-fat diet increased the carbonylated proteins (protein oxidative damage) in plasma by 45%, whereas supplementation with WKBE significantly reduced the protein carbonylation in plasma to levels similar to those observed in the control group [34]. Similar effects of WKBE on protein carbonylation were also observed in the heart tissue of mice, but these did not reach statistical significance [34]. Furthermore, WKBE increased the levels of two cardiac antioxidant enzymes, catalase reductase and glutathione reductase, but not nicotinamide adenine dinucleotide hydrogen (NADH) dehydrogenase, and prevented lipid infiltration in the aortic tissue. Although not statistically significant, there was also a trend for a reduction in high-fat diet-induced protein carbonylation as an indicator of cardiac oxidative stress. The findings from this study in a model of metabolic syndrome suggest that WKBE is able to attenuate some of the detrimental effects of this disease associated with high-fat diet-induced cardiovascular oxidative damage, and therefore further research into WKBE as a potential preventative strategy for metabolic syndrome and its co-morbidities are warranted.

In a rat model of diabetes, which is frequently associated with cardiovascular complications due to a greater number of reactive oxygen species and an impaired antioxidant system, Oliveira et al. [40] evaluated the effects of WKBE on oxidative stress-induced damage and collagen deposition in cardiac tissue. The treatment of diabetic rats with 100, 500 and 1500 mg/kg WKBE for 20 days increased the total antioxidant status of cardiac tissue and reduced the markers of oxidative stress (superoxidase dismutase and catalase activity) and concentrations of malondialdehyde (only with highest dose) when compared with untreated diabetic rats [40]. These effects of WKBE were accompanied by reduced collagen deposition, which was increased in the hearts of untreated diabetic rats. The authors of this study concluded that WKBE exerted anti-hyperglycemic effects against diabetes-induced oxidative stress and its consequential outcomes, e.g., collagen deposition, which may be protective against CVDs. However, further investigations are warranted to investigate the potential cardioprotective effects of WKBE. 

## 4. Evidence from Human Studies

Several human studies, typically conducted in overweight or obese individuals, have explored the effect of WKBE on weight loss and other body composition measurements, with supplement doses typically ranging from 445 to 3000 mg/d, study durations ranging from 28 to 84 days, and sample sizes ranging from 10 to 120 participants (Table 2). The majority of studies were randomised, double-blind placebo-controlled trials (RCTs), with some setting a daily calorie intake for participants or complementing with a multi-component weight-loss program [48,49,50]. The average weight loss from the human studies discussed was 2.6 kg and ranged from 1.8 to 3.5 kg.

### 4.1. Effects of WKBE on Body Weight and Composition

A change in body weight following the consumption of WKBE is the most common primary outcome reported in the literature. The earliest study exploring the effects of a commercially available WKBE on body weight was by Thom [50], who conducted a RCT in 40 overweight or obese but otherwise healthy participants. The participants were supplemented with two supplemental tablets (each containing ~200 mg WKBE, 200 mg inulin and 50 mg *Garcinia cambogia* extract) or a placebo after each meal, three times per day for 84 days. During this time, participants were also instructed to follow a low-fat diet comprising 1200 kcals/d. Those consuming the supplement containing WKBE significantly reduced their body weight (−3.5 kg), body fat percentage (−2.3%) and body mass index (BMI; −1.3 kg/m^2^) when compared with the baseline [50]. In contrast, there were no significant effects of the placebo on body weight (−1.3 kg), body fat (−0.7%) or BMI (−0.5 kg/m^2^), nor significant changes in the waist or hip circumference in either group. Nevertheless, given the presence of other bioactive ingredients in the supplement, this study did not allow the isolated effects of WKBE to be explored.

More recent studies have increased the dosage of WKBE, with the majority of studies administering >3000 mg/day, consumed in doses of ~1000 mg three times/day. In line with the findings observed by Thom [50], an 84-day RCT comprising 88 overweight participants reported a significant reduction in body weight in participants supplemented with 3 × 1000 mg/d WKBE (−3.1 kg) compared with those given the placebo (+0.36 kg) [51]. However, no significant changes in body fat, lean body mass or waist and hip circumferences were observed. In another study of the same duration, a greater weight loss was achieved in overweight or obese participants randomised to 3000 mg/d of a commercially available WKBE (IQP-PV-101, Phase 2, Starchlite and PhaseLite) versus the placebo (−2.9 vs. −0.9 kg, respectively). Similarly, WKBE resulted in greater reductions than the placebo in BMI (−1.1 vs. −0.3 kg/m^2^, respectively), fat mass (−2.2 vs. −0.65 kg, respectively) and waist circumference (−2.5 vs. −0.9 cm, respectively) at 84-days [52]. All participants in this study were on a hypocaloric diet, consuming 500 calories less than their basal energy needs per day, with 40% of daily energy intake from carbohydrates, demonstrating the potential complementary role of WKBE during a standard weight-loss regimen.

In another 84-day study, 62 overweight and obese participants consumed two capsules containing 150 mg WKBE (Wellex, LexMed ASA, Bergen, Norway) or a placebo three times per day, 30 min before each meal [53]. At the follow-up, participants randomised to WKBE had a significant reduction in body weight (−3.2 kg), BMI (−1.1 kg/m^2^), body fat (−2.8%) and waist circumference (−3.7 cm) when compared with the baseline. In contrast, there were no observed changes in body composition measurements in the placebo group. Following the first intervention, Birketvedt et al. [54] conducted an open-label trial for 252-days where the active participants (*n* = 24) were divided into a low-dose (two 150 mg capsules, three times per day) or a high-dose (four 150 mg capsules, three times per day) group. During the 252-day follow-up, body composition continued to improve (reductions in BMI, waist circumference, body weight, body fat) in both the low and high dose groups, and this was the first study to report a prolonged longer-term effect of WKBE supplementation on weight loss, without changes to lifestyle, for 365-days. However, no significant differences between the two doses used were observed, raising the interesting possibility that, with prolonged supplementation, low-dose WKBE—which could be more acceptable to users—may be equally as effective as higher doses. However, this notion requires further exploration, given this study did not have a control group during the 252-day open-label phase. 

Shorter-term studies investigating the effects of WKBE on weight loss, generally 56-days in duration, have been conducted. Wu et al. [55] randomised 101 healthy participants to consume either 1000 mg WKBE (Phase 2 Starch Neutralizer lV) or a placebo three times/day before each meal. Body weight and waist and hip circumference were reported at the baseline, 30 days in and the end of the intervention (60 days). The participants randomised to WKBE lost, on average, −1.9 kg post-intervention, compared with the placebo group, who lost −0.4 kg. Those supplemented with WKBE achieved a greater reduction in waist circumference (−1.9 cm vs. −0.4 cm for WKBE and placebo groups, respectively), although the hip circumference was unchanged. In the smallest study conducted in humans, 10 healthy participants with body fat ratios over 25% and over 30% for men and women, respectively, consumed 750 mg of WKBE per day (3 × capsules of Phaseolamin™ 1600 diet (Phase 2), twice a day, 30 min before lunch and dinner) for 56-days [56]. The participants achieved a significant reduction in body weight, body fat and BMI. Their waist and hip circumferences also decreased post-intervention when compared with the baseline, but there were no significant effects on the waist:hip ratio. It must be noted that the caloric intake decreased significantly over the 56-day intervention by approximately 200 kcals per day, suggesting that weight loss could have been a consequence of the reduction in energy intake as opposed to the WKBE alone.

In contrast, Udani et al. [57] reported no significant difference in weight loss in 27 obese participants randomised to 3000 mg/d WKBE (2 × 1500 mg/d) for 56-days compared with those given a placebo (−3.8 lbs vs. −1.7 lbs, respectively) [57]. Likewise, there was no significant effect of WKBE on body fat and waist circumference, although, as with weight loss, the reduction in body fat (−0.5%) and waist circumference (−1.5 inches) tended to be greater compared with the placebo group, for whom these values were unchanged [57]. Similar findings were reported by this group in a later, shorter study, where 2000 mg/d WKBE did not enhance weight loss or changes in waist circumference in healthy participants on a multi-component weight-loss programme for 28-days (−6.0 lbs vs. −4.7 lbs, respectively) [48]. However, interestingly, further exploratory analyses revealed that participants randomised to WKBE in the highest carbohydrate intake tertile experienced greater weight loss compared with individuals with a similar carbohydrate intake in the placebo group (WKBE −8.7 lbs vs. placebo −1.7 lbs). This suggests that WKBE may be a particularly effective weight-loss tool against the background of a high carbohydrate diet, where inhibiting carbohydrate digestion and absorption may have a more pronounced effect on overall energy intake compared with a lower carbohydrate diet. Furthermore, in a study performed in 60 weight-stable but overweight (by 5–15 kg) participants, a lower dose of WKBE (445 mg/day of Phase 2 Starch Neutralizer lV) consumed alongside a carbohydrate-rich diet was associated with significant reductions in body weight, BMI, fat mass, adipose tissue thickness and the waist:hip ratio whilst maintaining lean body mass when compared with the placebo—Celleno et al. [49].

In the most recent study, Wang et al. [58] reported that obese participants supplemented with 2400 mg WKBE/day achieved a significant loss of weight and fat mass by, on average, −2.2 kg and −2.0 kg, respectively, following a 35-day intervention; this differed significantly from that reported in the placebo group. The reductions in the overweight percentage, BMI and body fat percentage were also significantly greater in the treatment group vs. the placebo group. The subcutaneous fat thickness measured at the triceps, subscapular, abdomen and suprailiac, as well as circumferences of the waist and hip, all reduced significantly with WKBE supplementation, but not with the placebo.

The results from human studies suggest that WKBE may represent a useful alternative or adjunct to traditional weight-loss strategies, particularly when higher supplement doses and longer supplementation periods are prescribed. Moreover, the results from Udani and Singh [48] suggest that individuals with a greater carbohydrate intake may experience more pronounced weight loss with WKBE, which could be due to a greater reduction in carbohydrate absorption and thus a decreased energy yield from consumed food. Collectively, compared with the findings of animal model investigations, the findings from human studies present a more convincing case for an anti-obesity effect of WKBE. 

### 4.2. Effects of WKBE on Cardiometabolic Markers

Although much of the research in humans to date has focussed on the impact of WKBE supplementation on weight loss and body composition, outcomes related to metabolic risk factors, including blood glucose, triglycerides, cholesterol and blood pressure, have also been measured. The effects of WKBE on these parameters will be briefly discussed below.

#### 4.2.1. Blood Markers

In a small study supplementing 10 healthy participants with WKBE (750 mg/day) for 56-days, Koike et al. [56] reported a significant reduction in serum triglycerides and HDL cholesterol [56]. However, there were no significant changes in blood glucose concentrations, total cholesterol or LDL cholesterol. Similarly, Wu et al. [55] reported no effects of WKBE on circulating concentrations of cholesterol, triglycerides and glucose between the baseline and the 28 and 56-day follow-ups.

In a short-term RCT where 25 healthy participants consumed 1000 mg/day of a WKBE, there were no improvements in triglycerides, fasting glucose or total cholesterol when compared with the baseline measurements or the placebo group [48]. Surprisingly, in an earlier study, where participants consumed 3000 mg/day of a WKBE for 56-days, the authors reported a trend for an improvement in triglyceride levels, with a decrease of −26 mg/dL and −8 mg/dL in the WKBE and placebo groups, respectively. However, no additional blood biochemistry markers, e.g., HbA1C, or the total cholesterol improved [57]. Following the research group’s previous two studies, Udani et al. [59] performed an extensive open-label, six-arm crossover study to investigate the effects of WKBE on the glycemic index of white bread. Thirteen healthy, normoglycemic participants with a normal BMI completed the study. During the active phase, the participants consumed white bread (50 g net carbohydrates) with butter and 1500, 2000 or 3000 mg of WKBE in either capsule form (taken immediately before the food) or powder form (mixed into the butter). The addition of 3000 mg of powdered WKBE to the butter reduced the glycemic index of the white bread by 34% when compared with no WKBE, but this was not evident in the lower dose. The findings from this study suggest that the incorporation of WKBE into foods may reduce the glycemic index when compared with capsules due to increased bioavailability, which has important clinical implications for the management of metabolic disorders, where blood glucose and insulin resistance are common.

In a similar manner, Vinson et al. [60] conducted two double-blind, crossover pilot studies to investigate the effects of WKBE on glucose absorption [60]. In the first study, 11 normoglycemic participants consumed three tablespoons of soybean oil margarine with or without 1500 mg of WKBE (Phase 2, Pharmachem Labs, Kearney, NJ, USA) with four large slices of white bread and 4 g of Sweet N’Low™. The peak postprandial blood glucose concentrations were lower, and the time taken for the blood glucose to return to normal was faster with WKBE (62 min) when compared with the controls (80 min). The area under the glucose vs. time curve was also 66% lower with WKBE, suggesting that WKBE inhibited the digestion and absorption of approximately two-thirds of the carbohydrates in bread. In the second study, seven participants were given a standardised meal after an overnight fast, with or without 750 mg of WKBE; however, no effects were observed on glucose absorption, likely due to the differences in dosage and smaller sample size.

The haematology results from the most recent human study, including the red blood cell count, white blood cell count, platelet count and haemoglobin, did not change significantly with WKBE supplementation for 35 days, nor did they differ significantly from those given the placebo [58]. Likewise, serum biochemical parameters, including the total serum protein, albumin, glucose, uric acid and creatinine, did not change and remained within the normal ranges for these markers after a 35-day WKBE intervention in obese subjects, demonstrating the safety of WKBE.

#### 4.2.2. Blood Pressure

To date, a small number of studies have investigated the effects of WKBE on blood pressure [50,54,56,58]. Improvements in systolic and diastolic blood pressures of −5.6 and −3.9 mmHg, respectively, were reported following a 84-day supplementation with WKBE compared with the placebo [54]. Similar findings were also observed in a shorter study (56-days) [56]. In contrast, Thom [50] reported no significant improvements in blood pressure following a 84-day WKBE intervention. In obese individuals, there were no significant changes to blood pressure or heart rate with 2400 mg/d WKBE supplementation for 35 days when compared with the placebo [58]. Although a limited number of studies suggest that WKBE may improve blood pressure, it is unclear if this is a direct effect of the WKBE or secondary to weight and body fat loss, and requires further investigation.

#### 4.2.3. Appetite and Hunger

A limited number of studies have assessed the effects of WKBE on appetite and hunger in humans. Udani et al. [57] did not observe any differences in changes in appetite control or hunger between those given the WKBE or placebo for 56-days. These findings were substantiated in a later, shorter study by the same group where 1000 mg per day of WKBE was provided for 28-days [48]. Nevertheless, given some reports of an anorexogenic effect of WKBE in rodent models, further exploration in humans may be warranted.

### 4.3. Adverse Effects of WKBE

There have been no significant side effects reported with WKBE supplementation for up to 84-days (RCTs) or up to 365-days in an open-label trial [48,49,51,56,57,58]. A short-term study (28-days) reported no adverse effects of WKBE on kidney and liver function or blood counts (including white blood cells, haemoglobin and platelets) [48]. Similar findings were reported by this research group in an earlier study, although one participant in the WKBE group reported more tension headaches and one participant randomised to the placebo reported abdominal side effects [57]. More recently, Udani et al. [59] assessed the impact of a WKBE in capsule or powdered form on GI symptoms and reported no significant effects on diarrhoea, flatulence, bloating or nausea. In a longer study, Rothacker [51] also reported no adverse events following WKBE supplementation.

In the most recent human study, blood markers, including white blood cell and platelet counts all as indicators of adverse health effects, were not affected by supplementation in 120 obese participants consuming 2400 mg WKBE per day for 35 days [58]. Wang et al. [58] also explored the additional potential measures of adverse effects, including electrocardiogram, chest X-ray, abdominal ultrasound examinations, and heart rate and oxygen consumption during steady-state cycle ergometry (5 min at 100 watts power on an exercise bike). There were no significant changes within nor between the WKBE and placebo groups following the 35-day intervention. In addition, tests on urine and stools were performed and the authors found no colour, trait, pH nor microscopic abnormalities in the faeces or urine of participants in the treatment group when compared with those given the placebo.

The inhibition of digestion and absorption of carbohydrates consequently alters the substrate delivery to the GI tract, specifically the bacteria residing in the GI, as demonstrated by the significant changes in composition and diversity indices following WKBE consumption (see gut microbiota section) [61], which has been suggested to increase methane, carbon dioxide and hydrogen, which have been linked with bloating, flatulence and diarrhoea [25]. However, the data presented here suggest that WKBE is well tolerated and safe for consumption, even at the higher dosages of 3000 mg/day and with longer-term supplementation.

## 5. Conclusions and Future Directions

The evidence presented in this review indicates that WKBE aids weight loss, inducing small (average −2.6 kg) but potentially meaningful reductions in body weight in humans on a short- to medium-term basis (28 to 84 days) compared with a placebo, particularly when consumed alongside a high-carbohydrate diet [48]. Moving forward, longer duration RCTs would be useful to help establish whether similar or greater effects are apparent with more protracted supplementation periods. The potential to combine WKBE with other bioactive food extracts has received only a small amount of attention to date (see, for example, [50]), and future studies may wish to explore the potential additive effects of WKBE alongside other bioactive compounds, such as those which may inhibit the activity of lipase, to simultaneously attenuate fat digestion and absorption. An additional avenue for future research could be to establish acceptable and effective vehicles for the provision of WKBE, such as cereal bars or incorporation into meals with a high carbohydrate content. Such studies will need to carefully consider the production methods to ensure that the viability and efficacy of the extract is not compromised. The effects of WKBE on blood glucose concentrations and metabolic control warrants further investigation, given the possible role this bioactive food extract could play in the prevention or management of diabetes and related conditions such as CVD. Moreover, animal studies suggest a potential beneficial effect of WKBE on the gut microbiota, which could have applications both in weight management and in the treatment of other conditions where gut microbial dysbiosis has been implicated (e.g., diabetes, dementia, CVD) [62].

## Figures and Tables

**Table 1:** Animal studies investigating the effects of WKBE on body composition, metabolic risk factors and the gut microbiota.

Study	Animals	Duration	Preparation and Other Ingredients	Diet	Intervention Groups	Effects of WKBE
Song et al. [38]	48 *male C57BL/6J* mice,	98 days	Phase 2 (ZeLang, Nanjing, China)	High fat diet (HFD; 45% fat) and low fat diet (LFD; 10% fat)	WKBE: 50 mg/kg/d WKBE + HFD HFD: no WKBE Control: LFD	↓ weight gain ↓ visceral fat ↓ food and calorie intake ↓ triacylglycerol ↓ total, HDL and LDL cholesterol ↓ serum adiponectin ↓ serum glucose ↓ serum insulin ↓ HOMA-IR index ↑ HOMA-IS index ↓ Firmicutes, ↑ Verrucomicrobia and Actenobacteria (phylum level) ↑ *Bifidobacterium*, *Lactobacillus* and *Akkermansia* (*genus* level) (*p* < 0.05 vs. HFD alone)
Qin et al. [29]	80 healthy Sprague-Dawley rats	90 days	Isolated in-house from common white kidney beans (Kunming, China)	Conventional diet	L: 1 g/kg.bw WKBE M: 2 g/kg.bw WKBE H: 4 g/kg.bw WKBE Control (no WKBE)	No effect on body weight No effect on food intake No effect on haematology markers, e.g., haemoglobin, white blood cell count, platelet count No effect on blood biochemistry, e.g., cholesterol, triglycerides, glucose (N.S. vs. control)
Neil et al. [34]	Experiment 1: 90 C57BL6/J mice	84–238 days	Isolated in-house from cooked white kidney bean seed (Colorado State University, Fort Collins, CO, USA)	Not specified	WKBE: 40 g per 100 g feed Obese (HFD, no WKBE) Control (LFD, no WKBE)	↓ weight gain ↓ subcutaneous and visceral lipid accumulation (*p* < 0.05 vs. HF)
	Experiment 2: 16 male NCI C57BL6/NCr mice	84 days			WKBE: 40 g WKBE per 100 g feed + HFD Control (HFD)	No effect on body weight or BMI No effect on feed efficiency ↓ subcutaneous, visceral, retroperitoneal, epidiymal fat mass ↑ caecal bacterial content ↑ A. muciniphila ↓ Firmicutes: Bacteroidetes No effects on total energy excreted in faeces (*p* < 0.05 vs. control)
Shi et al. [33]	45 male Sprague-Dawley rats, high fat diet-induced obesity	70 days	Isolated in-house from white common bean seeds (cultivar Longquanjiuli) (Pinzhen food Co, Haerbin, China)	HFD: 45% fat Basic diet (control)	L: 0.5% WKBE M: 1% WKBE H: 1.5% WKBE Obese (no WKBE) Control (no HFD or WKBE)	↓ body weight at 6 (H dose) and 10 weeks (M and H dose) ↓ food intake ↓ food efficiency ratio ↓ HFD-induced intra-abdominal fat accumulation (M and H doses) ↓ HFD-induced increase in serum triglycerides (M and H doses) ↓ HFD-induced increase in LDL cholesterol ↑ total SCFAs ↑ acetic acid (H dose), propionic and isobutyric acid (M and H dose) Altered β-diversity of obese rats (H dose) ↓ Firmicutes ↑ Bacteroidetes and ↓ Firmicutes:Bacteroiedtes 30 different OTUs with H dose WKBE- including ↑ *Bacteroides*, *Butyricicoccus*, *Blautia*, *Eubacterium* and ↓ *Lactobacillus* and *Ruminococcus* (*p* < 0.05 vs. obese)
Preuss et al. [47]	16 Sprague-Dawley rats	63 days	Formula containing 19% dry bean extract (seed—*P. Vulgaris L.*), 31% hibiscus extract, 31% L-arabinose, 12% gymnema extract, 6% green tea extract leaf and 1% apple extract (AdvoCare International, Carrollton, TX, USA)	Regular rat chow, water *ad libitum*	WKBE: 2 g (1 g twice daily) of formula in 4 mL water (until wk.5)/sucrose solution (wk.5–9) Control: 4 mL water (until wk.5)/sucrose solution (wk.5–9)	No effect on body weight ↓ systolic BP No effect on food intake ↓ blood glucose ↓ circulating sodium and chloride ↑ circulating potassium and total protein No effect on haematology markers, e.g., haemoglobin, white blood cell count, platelet count (*p* < 0.05 vs. control)
Micheli et al. [35]	36 *male C57BL/6* mice, HFD-induced metabolic syndrome	56 days	Extract containing α-Amylase inhibitor from common kidney bean (*P. Vulgaris L.*) (Beanblock^®^; Indena S.p.A., Milan, Italy)	HFD: 60% fat, 20% protein, 20% carbohydrate Standard diet (control): 18% fat, 24% protein, 58% carbohydrate	WKBE: 500 mg kg^−1^ Obese: (HFD, no WKBE) Control (normal diet)	↓ weight gain No effect on food intake ↓ total and LDL cholesterol ↓ plasma glucose ↓ insulin tolerance ↓ plasma insulin ↓ triglycerides No effect on ghrelin ↓ oxidative stress markers protein carbonylation (in plasma but not heart tissue) ↑ cardiac antioxidant enzymes (catalase reductase and glutathione reductase, but not NADH dehydrogenase) (*p* < 0.05 WKBE vs. HF alone)
Tormo et al. [37]	Non-diabetic (ND) and type 2 diabetic (T2D) (neonatal diabetes models n0-STZ and n5-STZ) male Wistar rats. *n = not specified	22 days	Isolated in-house from white beans (*P. Vulgaris L.*) (100 mg/kg body weight dissolved in 9 g NaCl/l) for 22 d to non-diabetic (ND) and type 2 diabetic (neonatal diabetes models n0-STZ and n5-STZ) male Wistar rats	Standard diet: 2.7% fat, 61.4% carbohydrate, 15.1% protein, 3.9% fibre	WKBE T2D: 100 mg/kg.bw/day dissolved in NaCl(9 g/L) T2D control: NaCl (9 g/L) daily. WKBE ND: 100 mg/kg.bw/day dissolved in NaCl(9 g/L) ND control: NaCl (9 g/L)	↓ weight gain (WKBE ND vs. ND control) ↓ blood glucose (WKBE vs. T2D and ND controls) No effect on plasma insulin ↓ food intake (WKBE vs. T2D and ND controls) ↓ weight gain in WKBE ND vs. ND control)
Tormo et al. [36]	12 adult male Wistar rats	Acute study Chronic study: 21 days (chronic)	Isolated in house from white kidney bean meal	Standard diet: 2.7% fat, 61.4% carbohydrate, 15.1% protein, 4% fibre	Acute oral administration: Starch load ± WKBE (50 mg/kg body weight) Chronic study: WKBE: 50 mg kg^−1^ per day Control: *9 g/L NaCl per day*	Acute effects: ↓ blood glucose No effect on plasma insulin (P<0.05 vs. control; no WKBE) Chronic effects (post 21 days): ↓ weight gain ↓ blood glucose No effect on plasma insulin ↓ food intake (*p* < 0.05 vs. control)
Oliveira et al. [30]	48 male Wistar rats, induced diabetes (streptozotocin)	20 days	Phase 2 (Phaseolamin) (Nanjing Well Chemical Corp., Ltd., Nanjing, China)	Standard extruded chow and water *ad libitum*	D100: 100 mg/kg WKBE D500: 500 mg/kg WKBE D1500: 1500 mg/kg WKBE DACA: 25 mg/kg acarbose NTD (non-treated diabetic): no WKBE ND: non-diabetic, no WKBE control	↓ body weight D100 and NTD vs. ND ↓ glycaemia (D100, D500 and D1500 vs. NTD) No effect on total cholesterol ↓ alkaline phosphatase (D1500 vs. NTD) ↓ serum urea (D500 and D1500 vs. NTD) ↑ total antioxidant status ↓ cardiac oxidative stress markers (superoxidase dismutase, catalase, malondialdehyde, D1500 vs. NTD) ↓ cardiac collagen deposition
Deglaire et al. [32]	64 Sprague-Dawley male rats, protein-free diet (PFD)	14 days	*P. Vulgaris L.* extract (powder removed from Starch Stopper capsules, Palmerston North, New Zealand)	*Protein free diets (PFD): 0.36, 0.45 and 0.59 crude protein/100g diet*	*0.4% WKBE (45 mg/day) + PFD* *1.1% WKBE (120 mg/day) + PFD* *Control (PFD, no WKBE)*	No effect on body weight No effect on food intake (vs. all groups)
Preuss et al. [31]	96 Sprague-Dawley rats	Acute effects (one day)	Dry WKBE (AdvoCare International, Carrollton, TX, USA)	Regular rodent chow and water *ad libitum*	WKBE: 1 g WKBE (2 mL of water containing 0.5 g × 2, prior to and post CHO challenge) Control: no WKBE	↓ blood glucose above baseline levels (*p* < 0.05 vs. control)
	2 Yorkshire pigs		Capsule containing 19% dry bean extract (seed—*P. Vulgaris L.*), 31% hibiscus extract, 12% gymnema extract, 6% green tea extract leaf and 1% apple extract (AdvoCare International, Carrollton, TX, USA)	Food and water *ad libitum*	Crossover design: WKBE: 4 capsules + 200 g sucrose and/or rice starch in water Control: 200 g sucrose and/or rice starch in water	↓ blood glucose above baseline levels (*p* < 0.05 vs. control)
Carai et al. [39]	Experiment 1: 21 adult male Zucker *fa/fa* rats	3 treatments, 5 days each, 20 day wash-out periods in-between	Isolated in-house from common white kidney bean	Standard rat chow: 60% carbohydrate, 4% fibre, 16% protein, 3% fat	Control: 0 mg/kg WKBE 50 mg/kg WKBE 500 mg/kg WKBE suspended in distilled water +0·5 % methylcellulose and administered orally (2 mL/kg infusion volume)	↓ food intake ↓ body weight (*p* < 0.05 vs. control)
	Experiment 2: 15 adult male Zucker *fa/fa* rats	Acute effects		Standard rat chow: 60% carbohydrate, 4% fibre, 16% protein, 3% fat	0 mg/kg WKBE 50 mg/kg WKBE 500 mg/kg *WKBE* administered orally by 60 min before food presentation (2 mL/kg infusion volume)	↓ glycemia (*p* < 0.05 vs. control)

↓: reduced; ↑: increased, H: high dose; HFD: high fat diet; HOMA-IS: homeostasis model assessment of insulin sensitive index; M: medium dose; L: low dose; LFD: low fat diet; LPD: low protein diet; NADH: Nicotinamide adenine dinucleotide hydrogen; ND: non-diabetic; T2D: type 2 diabetic; WC: waist circumference.

**Table 2:** Human studies investigating the effects of WKBE on body composition and metabolic risk factors.

Study	Design	Duration	Dose, Preparation and Other Ingredients	Participants	Effects of WKBE
Birketvedt et al. [53] and [54]	RCT	90 days	Wellex capsules (LexMed ASA)- 900 mg WKBE per day	62 overweight/obese (BMI >25 kg/m^2^)	↓ body weight ↓ BMI ↓ body fat % ↓ WC ↓ systolic and diastolic BP ↓ total cholesterol No effect on HDL, LDL, triglycerides No effects on serum lipids or nutritional parameters (*p* < 0.05 vs. baseline)
Grube et al. [52]	RCT	84 days	3000 mg/day Phase 2 capsules	117 overweight/obese (BMI 25–35 kg/m^2^)	↓ body weight ↓ body fat mass ↓ WC (*p* < 0.001 vs. placebo)
Rothacker [51]	RCT	84 days	3000 mg/day Phase 2 capsules	88 overweight/obese (BMI 24–32 kg/m^2^)	↓ body weight (*p* < 0.05 vs. placebo) No effect on body fat No effect on lean body mass No effect on WC No effect on HC
Thom [50]	RCT	84 days	1200 mg/day Phase 2 capsules	40 overweight/obese (BMI 28–39 kg/m^2^)	↓ body weight ↓ BMI ↓ body fat % (*p* < 0.05 vs. baseline) No effect on WC No effect on HC No effect on BP
Wu et al. [55]	RCT	60 days	3000 mg/day Phase 2 capsules	101 overweight/obese (BMI 25–40 kg/m^2^)	↓ body weight ↓ waist circumference (*p* < 0.001 vs. placebo) No effect on HC No effect on blood biochemistry markers, e.g., cholesterol, triglycerides, blood glucose, creatinine, uric acid, apoliproteins
Udani et al. [57]	RCT	56 days	3000 mg/day Phase 2 capsules	27 obese (BMI 30–43 kg/m^2^)	No effect on body weight No effect on body fat No effect on WC Trend for reduction in triglycerides (*p* = 0.07) No effect on blood biochemistry markers, e.g., HbA1C, total cholesterol No effect on appetite control, hunger or energy levels (vs. placebo)
Koike et al. [56]	Open	56 days	750 mg/day Phase 2 capsules	10 (BMI 23–30 kg/m^2^, body fat >25% men and >30% women)	↓ body weight ↓ BMI ↓ body fat % ↓ waist circumference ↓ hip circumference ↓ triglycerides ↓ HDL cholesterol ↓ systolic and diastolic BP (*p* < 0.01 vs. baseline) No effect on waist:hip ratio No effect on blood glucose No effect on total or LDL cholesterol
Wang et al. [58]	RCT	35 days	2400 mg/day WKBE capsules (Yunnan Tianbaohua Biological Resources Development)	120 obese	↓ body weight ↓ BMI ↓ body fat % ↓ fat mass ↓ overweight (%) ↓ subcutaneous fat thickness (triceps, subscapular, abdomen, suprailiac) ↓ waist circumference ↓ hip circumference (*p* < 0.01 vs. baseline) No effect on BP No effect on blood biochemistry markers, e.g., glucose, albumin, uric acid, creatinine No effects on haematological markers, e.g., haemoglobin, red blood cell count, white blood cell count
Celleno et al. [49]	RCT	30 days	445 mg/day Phase 2 capsules, with carbohydrate-rich diet	60 overweight by 5–15 kg	↓ body weight ↓ BMI ↓ body fat % ↓ adipose tissue thickness ↓ waist circumference ↓ hip circumference ↓ right thigh circumference (*p* < 0.001 vs baseline and vs placebo)
Udani and Singh [48]	RCT	28 days	2000 mg/day Phase 2 capsules (plus multi-component weight-loss program)	25 healthy (BMI 23–31 kg/m^2^)	↓ body weight ↓ waist circumference (*p* < 0.01 vs. baseline, but N.S. vs. placebo) No effect on fasted glucose No effect on triglycerides No effect on total cholesterol No effect on appetite control, hunger or energy levels
Udani et al. [59]	Open, 6-arm crossover		1500, 2000 and 3000 mg WKBE in capsule and powder (incorporated into butter) form (consumed with white bread)	13 healthy normoglycemic (BMI 18–25 kg/m^2^)	↓ glycemic index of white bread
Vinson et al. [60]	Double-blind, crossover		1500 mg Phase 2 capsules (consumed with 4 large slices of white bread)	11 healthy normoglycemic	↓ peak postprandial blood glucose ↓ time of blood glucose normalisation (*p* < 0.05 vs. control)
	Double-blind, crossover		750 mg Phase 2 capsules	7 subjects	No effects on glucose absorption

↓: reduced; ↑: increased; BMI: body mass index; BP: blood pressure; HC: hip circumference; RCT: randomised controlled trial; WC: waist circumference; WKBE: white kidney bean extract.
